# The Relative Body Weight Gain From Early to Middle Life Adulthood Associated With Later Life Risk of Diabetes: A Nationwide Cohort Study

**DOI:** 10.3389/fendo.2022.927067

**Published:** 2022-07-19

**Authors:** Min Xu, Yan Qi, Gang Chen, Yingfen Qin, Shengli Wu, Tiange Wang, Zhiyun Zhao, Yu Xu, Mian Li, Li Chen, Lulu Chen, Yuhong Chen, Huacong Deng, Zhengnan Gao, Yanan Huo, Qiang Li, Chao Liu, Zuojie Luo, Yiming Mu, Guijun Qin, Feixia Shen, Lixin Shi, Qing Su, Qin Wan, Guixia Wang, Shuangyuan Wang, Youmin Wang, Ruying Hu, Yiping Xu, Li Yan, Tao Yang, Xuefeng Yu, Yinfei Zhang, Tianshu Zeng, Xulei Tang, Zhen Ye, Jiajun Zhao, Yufang Bi, Guang Ning, Jieli Lu, Weiqing Wang

**Affiliations:** ^1^ Department of Endocrine and Metabolic Diseases, Shanghai Institute of Endocrine and Metabolic Diseases, Ruijin Hospital, Shanghai Jiao Tong University School of Medicine, Shanghai, China; ^2^ Shanghai National Clinical Research Center for metabolic Diseases, Key Laboratory for Endocrine and Metabolic Diseases of the National Health Commission of the People's Republic of China, Shanghai Key Laboratory for Endocrine Tumor, State Key Laboratory of Medical Genomics, Ruijin Hospital, Shanghai Jiao Tong University School of Medicine, Shanghai, China; ^3^ Department of Endocrinology, Fujian Provincial Hospital, Fujian Medical University, Fuzhou, China; ^4^ Department of Endocrinology, The First Affiliated Hospital of Guangxi Medical University, Nanning, China; ^5^ Department of Endocrinology, Karamay Municipal People’s Hospital, Xinjiang, China; ^6^ Department of Endocrinology, Qilu Hospital of Shandong University, Jinan, China; ^7^ Department of Endocrinology, Union Hospital, Tongji Medical College, Huazhong University of Science and Technology, Wuhan, China; ^8^ Department of Endocrinology, The First Affiliated Hospital of Chongqing Medical University, Chongqing, China; ^9^ Department of Endocrinology, Dalian Municipal Central Hospital, Dalian, China; ^10^ Department of Endocrinology, Jiangxi Provincial People’s Hospital Affiliated to Nanchang University, Nanchang, China; ^11^ Department of Endocrinology, The Second Affiliated Hospital of Harbin Medical University, Harbin, China; ^12^ Department of Endocrinology, Jiangsu Province Hospital on Integration of Chinese and Western Medicine, Nanjing, China; ^13^ Department of Endocrinology, Chinese People’s Liberation Army General Hospital, Beijing, China; ^14^ Department of Endocrinology, The First Affiliated Hospital of Zhengzhou University, Zhengzhou, China; ^15^ Department of Endocrinology, The First Affiliated Hospital of Wenzhou Medical University, Wenzhou, China; ^16^ Department of Endocrinology, Affiliated Hospital of Guiyang Medical College, Guiyang, China; ^17^ Department of Endocrinology, Xinhua Hospital Affiliated to Shanghai Jiao Tong University, School of Medicine, Shanghai, China; ^18^ Department of Endocrinology, The Affiliated Hospital of Southwest Medical University, Luzhou, China; ^19^ Department of Endocrinology, The First Hospital of Jilin University, Changchun, China; ^20^ Department of Endocrinology, The First Affiliated Hospital of Anhui Medical University, Hefei, China; ^21^ Institute of Chronic Diseases, Zhejiang Provincial Center for Disease Control and Prevention, Hangzhou, China; ^22^ Clinical Trials Center, Ruijin Hospital Affiliated to Shanghai Jiao Tong University School of Medicine, Shanghai, China; ^23^ Department of Endocrinology, Sun Yat-sen Memorial Hospital, Sun Yat-sen University, Guangzhou, China; ^24^ Department of Endocrinology, The First Affiliated Hospital of Nanjing Medical University, Nanjing, China; ^25^ Department of Endocrinology, Tongji Hospital, Tongji Medical College, Huazhong University of Science and Technology, Wuhan, China; ^26^ Department of Endocrinology, Central Hospital of Shanghai Jiading District, Shanghai, China; ^27^ Department of Endocrinology, The First Hospital of Lanzhou University, Lanzhou, China; ^28^ Department of Endocrinology, Shandong Provincial Hospital, Jinan, China

**Keywords:** Body weight gain, early life adulthood, prospective cohort, type 2 diabetes, later life risk, obesity

## Abstract

**Aim:**

To determine the effect of decade-based body weight gain from 20 to 50 years of age on later life diabetes risk.

**Methods:**

35,611 non-diabetic participants aged ≥ 50 years from a well-defined nationwide cohort were followed up for average of 3.6 years, with cardiovascular diseases and cancers at baseline were excluded. Body weight at 20, 30, 40, and 50 years was reported. The overall 30 years and each 10-year weight gain were calculated from the early and middle life. Cox regression models were used to estimate risks of incident diabetes.

**Results:**

After 127,745.26 person-years of follow-up, 2,789 incident diabetes were identified (incidence rate, 2.18%) in 25,289 women (mean weight gain 20-50 years, 7.60 kg) and 10,322 men (7.93 kg). Each 10-kg weight gain over the 30 years was significantly associated with a 39.7% increased risk of incident diabetes (95% confidence interval [CI], 1.33-1.47); weight gain from 20-30 years showed a more prominent effect on the risk of developing diabetes before 60 years than that of after 60 years (Hazard ratio, HR = 1.084, 95% CI [1.049-1.121], *P <*0.0001 vs. 1.015 [0.975-1.056], *P* = 0.4643; *P*_Interaction_=0.0293). It showed a stable effect of the three 10-year intervals weight gain on risk of diabetes after 60 years (HR=1.055, 1.038, 1.043, respectively, all *P* < 0.0036).

**Conclusions:**

The early life weight gain showed a more prominent effect on developing diabetes before 60 years than after 60 years; however, each-decade weight gain from 20 to 50 years showed a similar effect on risk developing diabetes after 60 years.

## Introduction

Compelling evidence has demonstrated that overweight and obesity are major established and modifiable risk factors for cardiovascular risk, such as type 2 diabetes (T2D) (1–3). Majority of the studies focused on body weight close to the time of the disease diagnosis, usually the middle-aged and elderly life. Accumulating evidence supported that early adulthood weight could affect subsequent T2D risks regardless of weight close to the time of diagnosis, which suggested a longer history of relative overweight, starting earlier in life, poses an additional risk for developing diabetes (4–7). Besides initial body weight, weight change during early adulthood has also been demonstrated to have an independent effect on subsequent diabetes and other cardio-metabolic risk (7–12).

In China, the largest increase in the prevalence of obesity was seen in men between the ages of 20 and 40 and in women between 30 and 40 (13), which was similar with many other countries. The rising trend of obesity in China, especially in young people, will undoubtedly increase the prevalence of chronic diseases, leading to poor cardiovascular health (13, 14). Given that a large segment of the population is starting to gain weight early in adulthood, after settling into an occupation or family life, which is contributing to the obesity epidemic, it is important to determine whether elevated body weight in earl-life adulthood, or during the transition from early to middle-life adulthood, contributes independently to the disease risk. However, less is known whether body weight gain during different periods in early to middle adult life, usually 20 to 50 years old, are differently related to risk of T2D and how much such weight changes influence the diagnosis age of diabetes.

In this follow-up investigation, we used data from a large sample size well-defined community-based nation-wide cohort in China to determine the effect of decade- based body weight gain from 20 to 50 years old on developing diabetes risk of the later life.

## Materials and Methods

### Study Population

The study participants were from the China Cardiometabolic Disease and Cancer Cohort (4C) Study, which was an ongoing multicenter, population-based cohort study investigating associations of glucose homeostasis with clinical outcomes, including diabetes, cardiovascular diseases (CVDs), cancers, and all-cause mortality (15, 16). Briefly, a total of 20 communities from seven general geographic regions in China were selected during 2011-2012 (**Supplementary Table S1**). Eligible men and women aged ≥40 years were identified from local resident registration systems and invited to the study by home visiting by the trained community health workers. At baseline, a standard questionnaire was used to collect the lifestyle factors, disease and medical history, etc. Anthropometry measurements, 2-hour oral glucose tolerance tests (OGTTs), blood and urine sampling were conducted. During 2014-2015, we performed the first round of follow-up examination.

**Figure 1:**
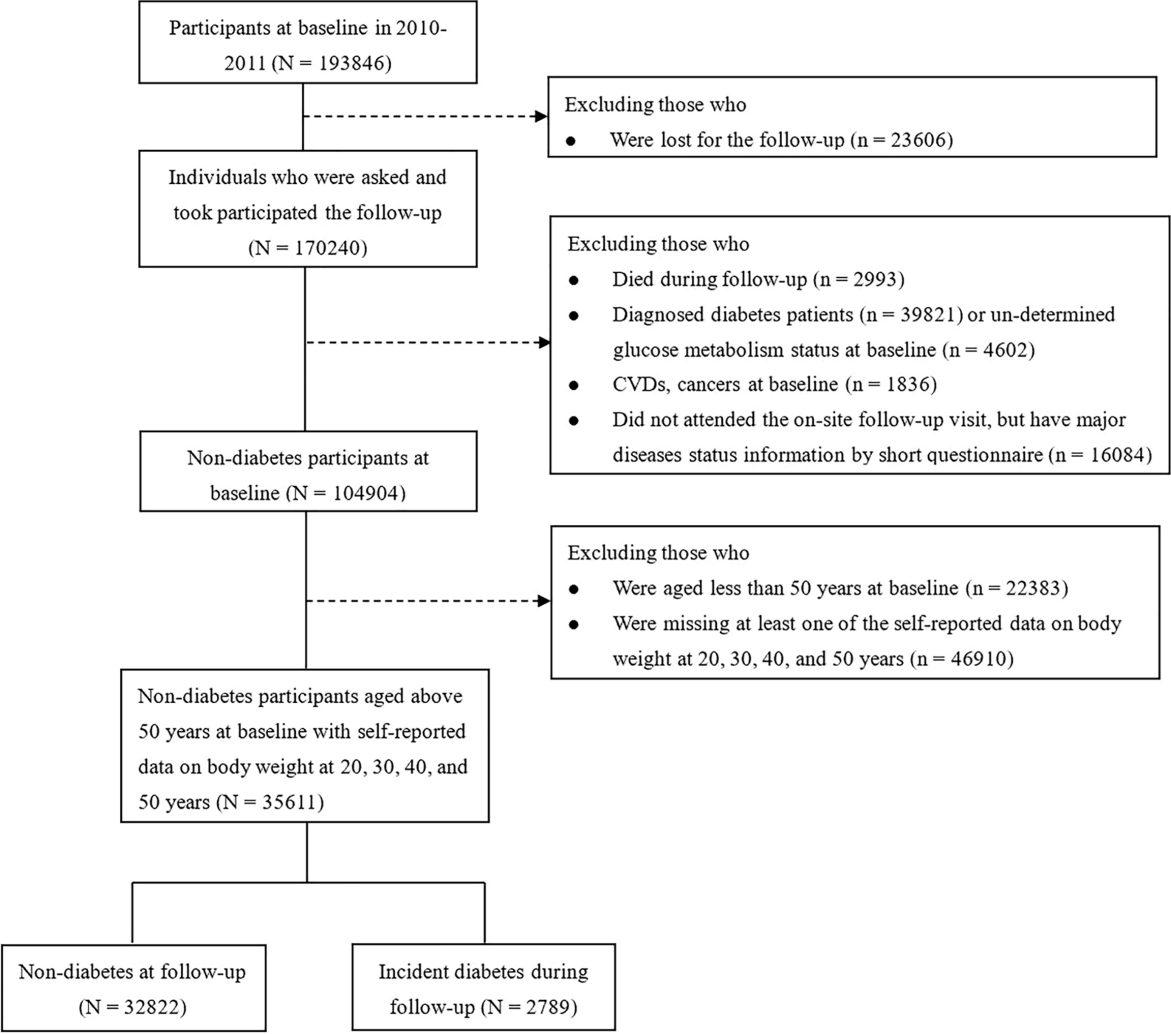
Flow chart of the present study.

A total of 193,846 individuals were recruited for the baseline and 170,240 participants attended the follow-up examination. In the present analysis, we excluded subjects who died during the follow-up (n=2,993); the baseline diagnosed diabetic patients (n=39,821) or un-determined glucose metabolism status (n=4,602); CVDs or cancers (n=1,836); or those who did not attend the on-site follow-up visit, but have major diseases status information by short questionnaire (n=16,084). For the specific aim of studying the effect of body weight during 20 to 50 years of age on the later life diabetes risk, we further excluded those people who were aged less than 50 years at baseline (n=22,383); or missing at least one of the self-reported data on body weight at 20, 30, 40 and 50 years (n=46,910). Thus, a total of 35,611 non-diabetic participants (25,289 women, 10,322 men) aged 50 years and above were finally included in the main analyses. The flow chart of participants selecting was described in **Figure 1**.

The study was approved by the Medical Ethics Committee of Ruijin Hospital, Shanghai Jiao-Tong University. Each participant provided written informed consent.

### Incident Diabetes Ascertainments

Incident T2D cases was defined according to the 2010 American Diabetes Association (ADA) criteria (fasting plasma glucose level ≥7.0 mmol/l or 2h OGTT glucose ≥11.1 mmol/l, or HbA1c ≥6.5%, or receiving glucose-lowering drugs or insulin injection) and/or a self-reported diagnosis by healthcare professionals.

### Exposure Assessment

Participants were asked to recall their body weight (kg) at their twenty, thirty, forty and fifty years old and record on the questionnaire. Weight gain over the 30 years (from 20 to 50 year of age) and decade-based weight gain were calculated by subtracting the self-reported body weight at the former time point from the one at the later time point. We assumed that body height is relatively stable during adulthood, and weight gain can represent one’s changes in adiposity during the early to middle life.

### Covariates Assessment

In the baseline questionnaires, we inquired information on risk factors for chronic diseases, such as cigarette smoking (current, former, or never smoker), and alcoholic intake, physical activity and sedentary time, education level (high school and above, or below high school) and family history of diabetes. Smoking status was defined as current, former, and never smokers. Current smokers were defined as smoking cigarettes every day or almost every day, with at least 7 cigarettes per week for at least 6 months. Former smokers were defined as cessation of smoking for more than 6 months. To assess the intensity of the smoking, the current/former smokers were used as two dummy variables, which was defined as the number of cigarettes each day, that were 0 cigarettes/day, 1–9 cigarettes/day, 10-19 cigarettes/day and ≥20 cigarettes/day. Never smokers were defined as participants who reported never smoked or had not smoked cigarettes regularly (<100 cigarettes in a lifetime). Alcohol intake was defined as drinker or non-drinker. Information regarding alcohol intake was collected by inquiring the information on the amount and type of alcoholic beverages consumed in the past 6 months, including beer, wine (e.g. red wine, or yellow wine, a typical wine made from rice and widely consumed in south China), and hard liquor. Beers are sold in 750-mL bottles, and wine and hard liquor in units of 0.5 kg. One unit of beer, wine, and liquor on average contains 30, 90, and 200 g ethanol, respectively. Average alcohol consumption was calculated by multiplying amount of alcohol consumed per drinking day by frequency (g/day). We used the Global Physical Activity Questionnaire to collect intensity, duration, and frequency of physical activity, the Metabolic Equivalent of Task (MET) minutes per week was used to measure physical activity level (17). Baseline body weight and height were measured according to a standard protocol by trained study staff. Baseline body weight and height were measured while they wore minimal clothing and no shoes, with values rounded to the nearest 0.1 kg and 0.1 cm, respectively. Body mass index (BMI) was calculated as body weight in kilograms divided by the square of body height in meters.

### Biochemical Measurements

Fasting and 2h post-loading blood sampling was performed during the OGTT. Participants were required to fast for ≥10 hours prior to filed examination. Plasma glucose concentrations were analyzed locally using a glucose oxidase or hexokinase method within two hours after blood sample collection under a stringent quality control program. Hemoglobin Capillary Collection System (Bio-Rad Laboratories, CA, USA) was used to collect finger capillary whole blood and shipped at 2-8°C to a certificated central laboratory at Ruijin Hospital. HbA1c was determined by high-performance liquid chromatography (VARIANT II System, Bio-Rad Laboratories, CA, USA). Fasting serum insulin was measured at the central laboratory using an auto-analyzer (ARCHITECT ci16200 analyzer, Abbott Laboratories, Illinois, USA). Homeostasis model assessment of insulin resistance (HOMA_IR) was calculated as fasting insulin × fasting plasma glucose/22.5, and beta cell function index (HOMA_β) was 20 × fasting insulin / (fasting plasma glucose − 3.5), where fasting insulin was in mIU/L and fasting plasma glucose in mmol/L.

### Statistical Analysis

Comparison of the baseline characteristics of the non-diabetic participants according to diabetes status at follow up examination by sex were conducted by ANOVA for continuous variables, and χ2 tests for categorical variables. The skewed distribution data were logarithmically transformed before statistical analysis.

We used Cox proportional hazards models to calculate multivariable-adjusted hazard ratios (HR) and 95% confidence intervals (CI) for the risk of T2D. For incident T2D, person-years were calculated from self-reported date of T2D diagnosis or the respective follow-up examination date for each individual or death or to end of follow-up (June 1, 2015), whichever came first. The following covariates were included in multivariable-adjusted models: age (continuous), sex, BMI at 20 years (continuous), baseline BMI (continuous), physical activity and sedentary time (in quintiles), smoking status (never, past, current), alcohol intake (g/day), high school and above (yes or no) and family history of diabetes (yes or no). The missing data were coded as a missing indicator category for categorical variables. We also tested the potential collinearity of BMI at 20 years age and BMI at baseline, and it showed that there was no collinearity of BMI at 20 years age and at baseline (the variance inflation factor, VIF was 1).

We tested interactions of age and other predictors of T2D by putting body weight gain in each 10 year from 20-50 years or the overall weight gain over the 30 years, each strata variable and the interaction terms (weight gain x the strata variable) in the same multivariate model. We also conducted stratified analysis according to baseline age (< or ≥60 years), sex, BMI at 20 years (< or ≥the median value, 21 kg/m^2^), baseline BMI (< or ≥25 kg/m^2^), physical activity (in quintiles), current smoking (yes or no).

All the analyses were carried out with SAS software, version 9.3 (SAS Institute), at a two-tailed alpha level of 0.05.

## Results

We determined 2,789 incident diabetes after a follow up of 127,745.26 person-years from the 35,611 non-diabetic participants aged ≥ 50 years. The incidence rate of T2D was 2.18%, 95% CI (2.10% - 2.17%), with 2.43% in men and 2.08% in women.

From early to middle adulthood, the mean weight gain from 20 to 50 years [SD] was: 7.60 [7.95] kg in women and 7.93 [8.54] kg in men; each 10-year weight gain from 20-30, 30-40, and 40-50 years were: 2.78 [4.88], 2.39 [4.26], and 2.42 [4.40] kg in women; and 2.67 [4.12], 2.76 [4.66], and 2.50 [4.92] kg in men, respectively.

Baseline characteristics of participants were summarized in **Table 1**. Consistently among total participants, men and women, those who developed diabetes had a higher body weight at 20 years, more weight gain from 20 to 50 years, as well as each-decade based weight gain (**Table 1**). Body weight at 20 years and each-decade weight gain from 20 to 50 years were independently and significantly associated with an increased risk of incident diabetes (**Supplementary Table S2**).

**Table 1:** Baseline characteristics of the non-diabetic participants according to sex and diabetes status at follow up examination.

	Total cohort			Men			Women		
	Non-diabetes	Diabetes	*P*	Non-diabetes	Diabetes	*P*	Non-diabetes	Diabetes	*P*
*n*. of participants	32,822	2,789		9,422	900		23,400	1,889	
Age at baseline, years	58.8 (6.7)	59.7 (6.7)	<.0001	60.0 (7.0)	60.2 (6.9)	0.4584	58.3 (6.5)	59.4 (6.6)	<.0001
BMI at baseline, kg/m^2^	24.5 (3.5)	25.6 (3.4)	<.0001	24.6 (3.4)	25.6 (3.2)	<.0001	24.5 (3.5)	25.6 (3.5)	<.0001
BMI at age of 20 y, kg/m^2^	21.2 (2.9)	21.3 (2.8)	0.0327	21.4 (2.6)	21.4 (2.5)	0.7896	21.2 (3.0)	21.3 (2.9)	0.0292
BMI at age of 30 y, kg/m^2^	22.3 (2.9)	22.7 (2.9)	<.0001	22.3 (2.6)	22.6 (2.5)	0.0003	22.3 (3.0)	22.7 (3.0)	<.0001
BMI at age of 40 y, kg/m^2^	23.2 (3.0)	23.9 (3.1)	<.0001	23.3 (2.9)	23.9 (2.9)	<.0001	23.2 (3.1)	23.9 (3.2)	<.0001
BMI at age of 50 y, kg/m^2^	24.2 (3.3)	25.1 (3,3)	<.0001	24.1 (3.2)	25.0 (3.1)	<.0001	24.2 (3.3)	25.1 (3.4)	<.0001
Height at baseline, cm	159.3 (7.6)	159.4 (7.7)	0.4918	166.5 (6.5)	166.3 (6.2)	0.4196	156.4 (5.8)	156.1 (5.9)	0.0371
Weight at age of 20 y, kg	53.8 (7.9)	54.2 (8.0)	0.0136	59.2 (7.3)	59.2 (7.6)	0.9391	51.7 (7.1)	51.8 (7.1)	0.2566
Each decade weight gain, kg									
20-30 y	2.49 (4.67)	3.44 (4.63)	<.0001	2.59 (4.09)	3.45 (4.32)	<.0001	2.73 (4.89)	3.43 (4.77)	<.0001
30-40 y	2.45 (4.36)	3.09 (4.69)	<.0001	2.70 (4.63)	3.43 (4.93)	<.0001	2.35 (4.24)	2.93 (4.56)	<.0001
40-50 y	2.39 (4.54)	3.04 (4.79)	<.0001	2.44 (4.90)	3.12 (5.12)	<.0001	2.38 (4.38)	3.00 (4.62)	<.0001
Glucose traits at baseline									
Fasting glucose, mmol/L	5.46 (0.51)	5.77 (0.57)	<.0001	5.50 (0.52)	5.78 (0.59)	<.0001	5.44 (0.51)	5.76 (0.55)	<.0001
OGTT 2h post-loading glucose, mmol/L	6.84 (1.62)	7.93 (1.79)	<.0001	6.65 (1.73)	7.78 (1.89)	<.0001	6.91 (1.56)	8.00 (1.73)	<.0001
HbA1c level, %	5.75 (0.36)	5.90 (0.36)	<.0001	5.70 (0.36)	5.87 (0.38)	<.0001	5.77 (0.35)	5.91 (0.36)	<.0001
HOMA_β, %	69.0 (49.5-95.9)	68.9 (46.5-96.6)	0.8177	58.4 (40.3-84.2)	59.6 (39.6-86.4)	0.6236	72.9 (53.7-100)	72.6 (51.0-101.7)	0.7730
HOMA_IR	1.60 (1.13-2.24)	2.02 (1.39-2.80)	<.0001	1.39 (0.94-2.02)	1.79 (1.20-2.50)	<.0001	1.68 (1.22-2.32)	2.11 (1.52-2.89)	<.0001
Physical activity, MET-h/wk									
Quintile 1	0	0	0.3773	0	0	0.0397	0	0	0.3452
Quintile 2	9.46 (2.93)	9.70 (2.81)		9.59 (2.87)	9.73 (2.63)		9.41 (2.95)	9.68 (2.90)	
Quintile 3	22.0 (2.65)	21.9 (2.71)		21.9 (2.69)	22.0 (2.62)		22.0 (2.64)	21.9 (2.74)	
Quintile 4	40.9 (7.11)	41.0 (7.06)		41.0 (7.14)	41.1 (7.23)		40.8 (7.10)	41.0 (6.99)	
Quintile 5	94.1 (51.9)	96.7 (61.4)		99.2 (55.9)	98.6 (54.8)		92.0 (55.0)	95.5 (65.0)	
Sedentary time, hours/day									
Quintile 1	1.45 (0.69)	1.50 (0.66)	0.0787	1.45 (0.69)	1.52 (0.63)	0.2043	1.46 (0.69)	1.49 (0.67)	0.1344
Quintile 2	2.87 (0.25)	2.91 (0.22)		2.86 (0.25)	2.92 (0.21)		2.87 (0.24)	2.90 (0.22)	
Quintile 3	3.87 (0.24)	3.89 (0.23)		3.87 (0.24)	3.89 (0.22)		3.88 (0.24)	3.89 (0.23)	
Quintile 4	4.97 (0.34)	4.93 (0.32)		5.00 (0.34)	4.96 (0.35)		4.96 (0.34)	4.92 (0.30)	
Quintile 5	7.35 (1.59)	7.37 (1.62)		7.45 (1.68)	7.66 (1.91)		7.30 (1.55)	7.22 (1.42)	
Alcohol consumption, grams/day									
0	29,334 (89.4)	2,458 (88.1)	0.0385	6,349 (67.4)	594 (66.0)	0.2743	22,985 (98.2)	1,864 (98.7)	0.4749
0-20	809 (2.46)	70 (2.51)		579 (6.15)	58 (6.44)		230 (0.98)	12 (0.64)	
20-40	720 (2.19)	57 (2.04)		639 (6.78)	51 (5.67)		81 (0.35)	6 (0.32)	
≥ 40	1,959 (5.57)	204 (7.31)		1,855 (19.7)	197 (21.9)		104 (0.44)	7 (0.37)	
Smoking status									
Never smoker	26,760 (82.7)	2,222 (80.2)	0.002	4,009 (43.1)	395 (44.2)		22,751 (98.6)	1,827 (97.6)	0.0003
Past smoker									
0	31,341 (95.5)	2,649 (94.9)	0.6265	8,027 (85.2)	771 (85.7)	0.7791	23,314 (99.6)	1,878 (99.4)	0.0330
0-10 cigarettes/day	374 (1.14)	35 (1.25)		315 (3.34)	27 (3.00)		59 (0.25)	8 (0.42)	
10-20 cigarettes/day	318 (0.97)	28 (1.00)		309 (3.28)	25 (2.78)		9 (0.04)	3 (0.16)	
≥ 20 cigarettes/day	77 (2.76)	77 (2.76)		771 (8.18)	77 (8.56)		18 (0.08)	0	
Current smoker									
0	28,691 (87.4)	2,385 (85.5)	0.0384	5,518 (58.6)	530 (58.9)	0.9314	23,173 (99.0)	1,855 (98.2)	0.0036
0-10 cigarettes/day	678 (2.07)	68 (2.44)		584 (6.20)	51 (5.67)		94 (0.40)	17 (0.90)	
10-20 pack-years	1,195 (3.64)	116 (4.16)		1,132 (12.0)	107 (11.9)		63 (0.27)	9 (0.48)	
≥ 20 cigarettes/day	2,258 (6.88)	220 (7.89)		2,188 (23.2)	212 (23.6)		70 (0.30)	8 (0.42)	
Education level									
High school and above	19,833 (60.4)	1,691 (60.6)	0.8316	5,545 (58.9)	498 (55.3)	0.0407	14,288 (61.1)	1,193 (63.2)	0.0722
Junior high school and below	12,989 (39.6)	1,098 (39.4)		3,877 (41.2)	402 (44.7)		9,112 (38.9)	696 (36.8)	
Family history of diabetes	4,032 (12.3)	434 (15.6)	<.0001	883 (9.37)	131 (14.6)	<.0001	3,149 (13.5)	303 (16.0)	0.0017

Data are presented as means ± standard deviation (SD), or medians (inter-quartile ranges) for skewed variables, or number (proportions) for categorical variables. P values were from the ANOVA without any adjustments. Abbreviations: IQR, inter quartile range; MET, metabolic equivalent task.

Among total cohort, each 10-kg weight gain over the 30 years period (20-50 years) was significantly associated with 39.7% increased risk of incident diabetes (95% CI, 1.33-1.47) (HR 1.37, 95% CI [1.26-1.49] in men; and 1.40 [1.32-1.49] in women) after adjustments for a full list of confounders. The corresponding HRs and 95%CI for each 1-kg/m^2^ weight gain from 20-50 years were 1.036, 95% CI 1.001-1.071, P = 0.0427 in men and 1.047, 95% CI 1.025-1.069, P <.0001 in women (**Table 2**). However, the associations were not significantly different between men and women (P for interaction = 0.286). The HR and 95% CI of T2D in weight gain (each 1-kg/m^2^ BMI) from 20-30 years was 1.055 (1.029-1.081), 1.038 (1.012-1.064) for weight gain form 30-40 years and 1.043 (1.017-1.069) for 40-50 years, respectively. In both sexes, the weight gain from 20-30 years showed a nominally higher risk of developing diabetes than the other two-decades interval; however, no significant interaction between sex and the different life stage weight gain was detected (all P for interaction > 0.344 in men, and 0.329 in women) (**Table 2**).

**Table 2:** Hazard risk of weight gain in different life stage with risk of incident diabetes in total and sex-specific samples.

		Model 1		Model 2	
		HR, 95% CI	*P*	HR, 95% CI	*P*
Total cohort					
Cases/Participants	2,789/35,611				
Person-years	127,745.26				
Incidence rate, 95% CI	2.18% (2.10%-2.17%)				
Weight gain, each 1-kg/m^2^					
from 20-30 years age		1.060 (1.035-1.085)	<.0001	1.055 (1.029-1.081)	<.0001
from 30-40 years age		1.042 (1.018-1.067)	<.0001	1.038 (1.012-1.064)	0.0036
from 40-50 years age		1.049 (1.024-1.074)	<.0001	1.043 (1.017-1.069)	0.0009
Weight gain, from 20-50 years age, each 1-kg/m^2^		1.050 (1.032-1.068)	<.0001	1.045 (1.027-1.064)	<.0001
Men					
Cases/Participants	900/10,322				
Person-years	37,108.18				
Incidence rate, 95% CI	2.43% (2.27%-2.59%)				
Weight gain, each 1-kg/m^2^					
from 20-30 years age		1.083 (1.032-1.137)	0.0011	1.071 (1.018-1.127)	0.0259
from 30-40 years age		1.026 (0.984-1.070)	0.2266	1.020 (0.975-1.067)	0.3911
from 40-50 years age		1.035 (0.992-1.080)	0.1137	1.027 (0.982-1.074)	0.2425
Weight gain, from 20-50 years age, each 1- kg/m^2^		1.044 (1.011-1.077)	0.0079	1.036 (1.001-1.071)	0.0427
Women					
Cases/Participants	1,889/25,289				
Person-years	90,637.08				
Incidence rate, 95% CI	2.08% (1.99%-2.18%)				
Weight gain, each 1-kg/m^2^					
from 20-30 years age		1.054 (1.026-1.083)	0.0001	1.051 (1.022-1.081)	0.0006
from 30-40 years age		1.048 (1.018-1.078)	0.0015	1.042 (1.010-1.074)	0.0085
from 40-50 years age		1.053 (1.024-1.083)	0.0003	1.048 (1.017-1.079)	0.0022
Weight gain, from 20-50 years age, each 1-kg/m^2^		1.052 (1.031-1.073)	<.0001	1.047 (1.025-1.069)	<.0001

Data are hazard ratio, 95% confidence interval (CI). P values were calculated from the Cox regression models. Model 1, adjusted for age, sex, BMI at 20 years age and BMI at baseline. Model 2, further adjusted for quintiles of physical activity, quintiles of sedentary time, smoking status (current, former, and never), alcohol drinking (g/day), and education level (high school and above or low), and diabetes family history (yes or no).

**Figure 2:**
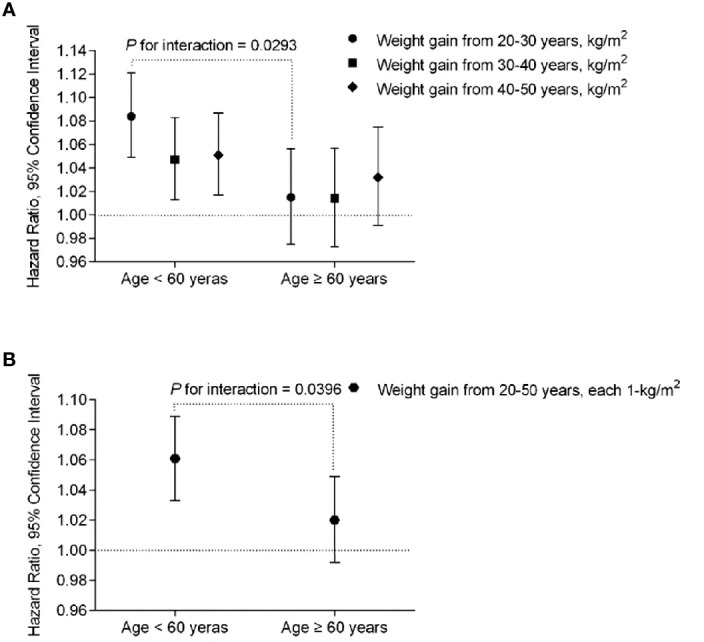
The risk of each 1-kg/m^2^ body weight gain during the 3-decade intervals from early to middle life with diabetes risk stratified by age < 60 or ≥ 60 years at baseline, respectively. The multivariable-adjusted hazard ratios (HR) and 95% confidence intervals (CI) for the risk of T2D were calculated by Cox proportional hazards models, after adjustments for age (continuous), sex, body height, BMI at 20 years (continuous), baseline BMI (continuous), physical activity and sedentary time (in quintiles), smoking status (never, past, current), alcohol intake (g/day), high school and above (yes or no) and family history of diabetes (yes or no). *P*_interactions_ were from the Cox proportional hazards models, by putting age and body weight gain in each 10 years from 20-50 years or the overall weight gain over the 30 years, and the interaction terms (weight gain x age) in the same multivariable-adjusted model, simultaneously. **(A)** Association of each-decade increase of body weight with risk of incident diabetes stratified by age < 60 or ≥ 60 years at baseline. **(B)** Association of total body weight gain from age 20-50 (each 1 kg/m2) with risk of incident diabetes stratified by age < 60 or ≥ 60 years at baseline.

We further performed stratified analysis according to age, BMI at 20 years and lifestyle factors at baseline. In the participants younger than 60 years, weight gain from 20-30 years showed more prominent effect on risk of developing diabetes than that in those older than 60 years (HR 1.084, 95% CI [1.049-1.121] vs. 1.015, 95% CI [0.975-1.056], *P*_interaction_=0.0293) (**Table 3**, **Figure 2A**). Same trend was found with regard to total weight gain over the 30 years (**Table 3**, **Figure 2B**). It showed a stable effect of the three 10-year intervals weight gain from 20 to 50 years on risk of developing diabetes after 60 years; however, the results did not remain significant after adjusted for the baseline BMI.

**Table 3:** Stratified analysis of the hazard risk of weight gain in different life with risk of incident diabetes.

		Model 1		Model 2	
		HR, 95% CI	*P*	HR, 95% CI	*P*
Age < 60 years					
Cases/Participants	1,613/22,585				
Person-years	80,388.40				
Incidence rate, 95% CI	2.01% (1.91%-2.11%)				
Weight gain, each 1-kg/m^2^					
from 20-30 years		1.083 (1.049-1.118)	<.0001	1.084 (1.049-1.121)	<.0001
from 30-40 years		1.053 (1.021-1.087)	0.0012	1.047 (1.013-1.083)	0.0067
from 40-50 years		1.055 (1.022-1.088)	0.0009	1.051 (1.017-1.087)	0.0032
Age ≥ 60 years					
Cases/Participants	1,176/13,026				
Person-years	47,356.86				
Incidence rate, 95% CI	2.48% (2.34%-2.63%)				
Weight gain, each 1-kg/m^2^					
from 20-30 years		1.023 (0.985-1.063)	0.2331	1.015 (0.975-1.056)	0.4643
from 30-40 years		1.017 (0.978-1.057)	0.4104	1.014 (0.973-1.057)	0.5094
from 40-50 years		1.041 (1.001-1.082)	0.0451	1.032 (0.991-1.075)	0.1286
BMI at baseline < 25kg/m^2^					
Cases/Participants	1215/20,346				
Person-years	72,978.38				
Incidence rate, 95% CI	1.67% (1.57%-1.76%)				
Weight gain, each 1-kg/m^2^					
from 20-30 years		1.071 (1.034-1.109)	0.0001	1.075 (1.036-1.116)	0.0001
from 30-40 years		1.063 (1.027-1.100)	0.0005	1.058 (1.020-1.097)	0.0027
from 40-50 years		1.065 (1.029-1.103)	0.0004	1.066 (1.028-1.106)	0.0006
BMI at baseline ≥ 25kg/m^2^					
Cases/Participants	1,556/15,004				
Person-years	53,807.52				
Incidence rate, 95% CI	2.89% (2.75%-3.04%)				
Weight gain, each 1-kg/m^2^					
from 20-30 years		1.065 (1.031-1.101)	0.0002	1.055 (1.020-1.092)	0.0022
from 30-40 years		1.040 (1.004-1.076)	0.0267	1.034 (0.998-1.072)	0.0677
from 40-50 years		1.045 (1.010-1.080)	0.0100	1.033 (0.997-1.069)	0.0700
Low BMI at 20 years					
Cases/Participants	1,313/17,330				
Person-years	63,539.47				
Incidence rate, 95% CI	2.07% (196%-2.18%)				
Weight gain, each 1-kg/m^2^					
from 20-30 years		1.031 (0.988-1.077)	0.1638	1.025 (0.979-1.073)	0.2846
from 30-40 years		1.005 (0.959-1.052)	0.8417	0.999 (0.950-1.050)	0.9642
from 40-50 years		1.004 (0.959-1.052)	0.8645	1.009 (0.961-1.060)	0.7144
High BMI at 20 years					
Cases/Participants	1,403/17,175				
Person-years	61,131.70				
Incidence rate, 95% CI	2.30% (2.18%-2.42%)				
Weight gain, each 1-kg/m^2^					
from 20-30 years		1.044 (1.011-1.078)	0.0077	1.042 (1.008-1.078)	0.0148
from 30-40 years		1.040 (1.008-1.074)	0.0142	1.035 (1.001-1.070)	0.0433
from 40-50 years		1.042 (1.010-1.075)	0.0092	1.032 (0.998-1.066)	0.0631
Never smoker					
Cases/Participants	2,222/28,982				
Person-years	103,916.14				
Incidence rate, 95% CI	2.14% (2.05%-2.23%)				
Weight gain, each 1-kg/m^2^					
from 20-30 years		1.059 (1.032-1.086)	<.0001	1.056 (1.028-1.085)	<.0001
from 30-40 years		1.048 (1.021-1.075)	0.0003	1.042 (1.014-1.070)	0.0034
from 40-50 years		1.041 (1.014-1.069)	0.0026	1.035 (1.007-1.064)	0.0152
Ever-smoker					
Cases/Participants	544/6156				
Person-years	22,155.32				
Incidence rate, 95% CI	2.46% (2.25%-2.67%)				
Weight gain, each 1-kg/m^2^					
from 20-30 years		1.048 (0.981-1.119)	0.1619	1.030 (0.962-1.103)	0.3983
from 30-40 years		1.006 (0.944-1.072)	0.8522	0.998 (0.934-1.067)	0.9580
from 40-50 years		1.058 (0.998-1.121)	0.0576	1.049 (0.988-1.115)	0.1194

Data are hazard ratio, 95% confidence interval (CI). P values were calculated from the Cox regression models. Model 1, adjusted for age, sex, BMI at 20 years age and BMI at baseline. Model 2, further adjusted for quintiles of physical activity, quintiles of sedentary time, smoking status (current, former, and never), alcohol drinking (g/day), and education level (percentage of high school and above), and diabetes family history (yes or no).

It also showed that weight gain in each-decade was significantly associated with risk of later life diabetes in participants with higher BMI at their 20 years (≥21 kg/m^2^); while no significant association was found in those with lower level (< 21 kg/m^2^) (**Table 3**). No significant interactions were found between body weight at 20 years, baseline BMI or the smoking status at baseline with the weight gains of each 10-year intervals on the risk of incident diabetes (all *P*_interaction_ ≥0.05). Characteristics of the participants by their BMI at 20 years and baseline BMI categories was shown in **Supplementary Table S3**.

**Figure 3:**
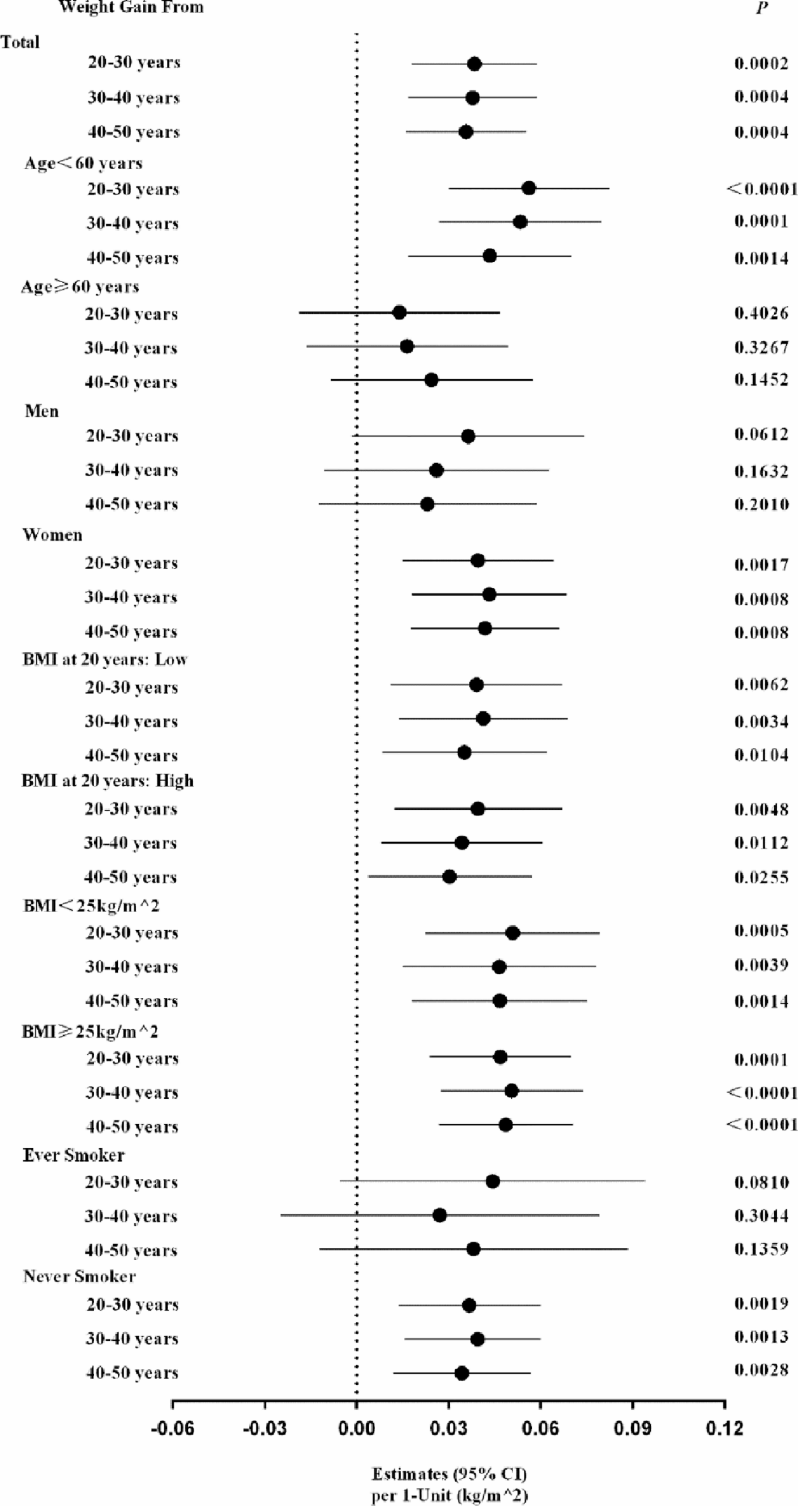
The estimated risk of each 1-kg/m^2^ of each-decade body weight gain from early to middle life with diabetes risk: data from the multiple imputation methods. The multiple imputation methods were conducted in dealing with the missing data in BMI at 20, 30, 40 and 50 years of age, and the above-mentioned covariates, using the Markov chain Monte Carlo (MCMC) method, to estimate the risk of weight gains on diabetes in total participants and each risk factors strata.

On considering a high proportion of the participants failed to report their body weight at least one time per year (44.7%, 46,910/104,904), we validated the robustness of the present analysis by conducting the multiple imputation analysis. We imputed missing data not only in BMI at 20, 30, 40 and 50 years of age, and but also the above mentioned covariates (n=82,521), using the Markov Chain Monte Carlo (MCMC) method, to estimate the risk ratio of weight gain on diabetes (SAS software, version 9.2, SAS Institute, Cary, North Carolina) (18). **Figure 3** shows the main and stratified analysis results using the procedure of MIANALYZE, which were consistent with that calculating from the previous procedure in which the missing data for weight at each time point were excluded.

## Discussion

**Figure 4:**
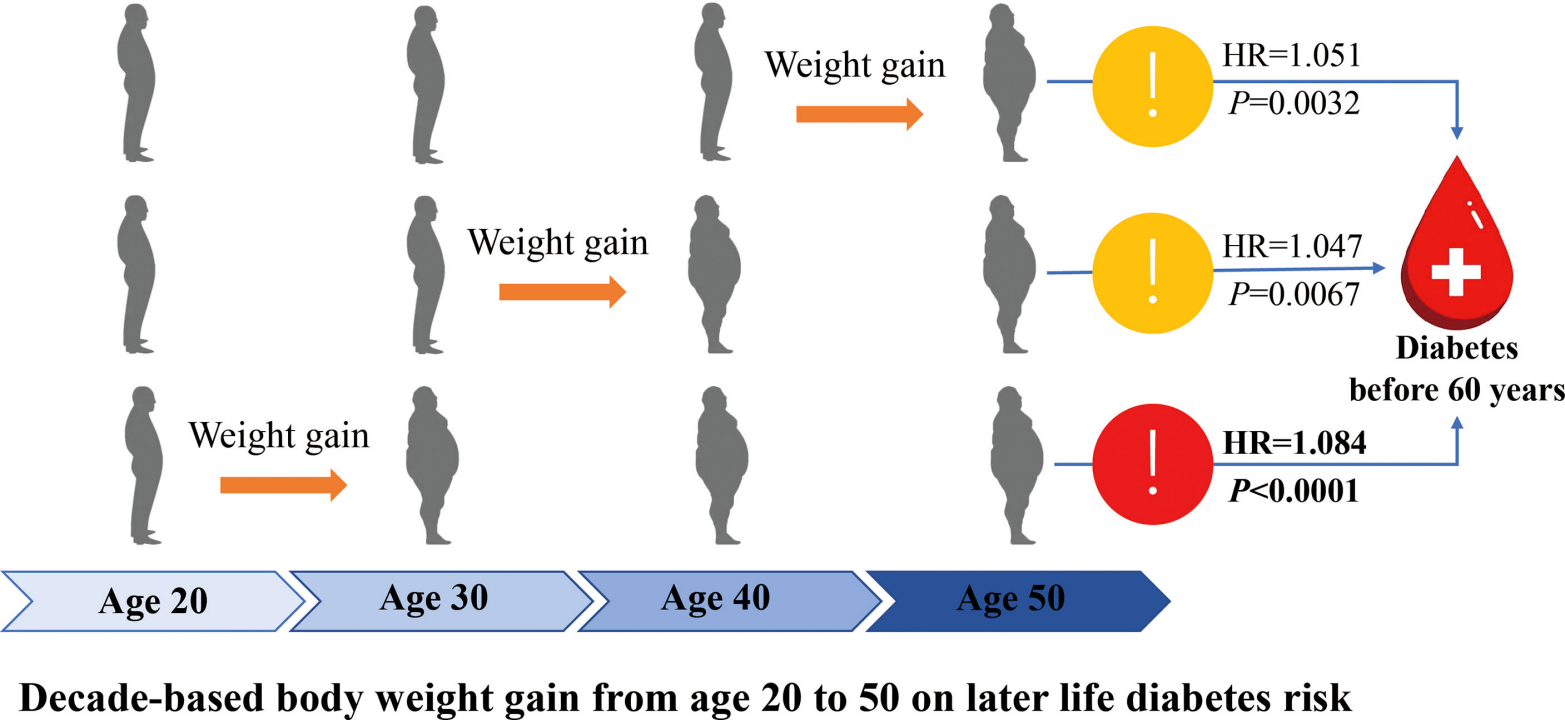
A graphic presentation of main findings of present study.

In the present large population-based nation-wide follow up study, we found that weight gain from early to middle life was significantly associated with increased later life diabetes risk. Weight gain in early life of the adulthood, usually 20 to 30 years showed more prominent effect on developing diabetes before 60 years than that for developing diabetes after 60 years; however, each-decade weight gain over the adulthood from 20 to 50 years showed similar effect on the risk of developing diabetes after 60 years. The main findings were summarized graphically in **Figure 4**.

The prevalence of obesity gradually increases from 20 to 60 years of old. A large percentage of T2D develops after 40 to 70 years (13, 19–21). Previous studies have demonstrated that besides initial body weight, weight change during early adulthood poses an independent role on subsequent diabetes risk (7, 12), including recent two large, long-term cohorts, which showed that weight change from early to middle adulthood (age of 18 or 21 to 55 years) was associated with increased risk of morbidity and mortality, independent of weight at early adulthood (11). In addition, we have summarized the scientific literature of similar research works as **Supplementary Table 4**. In the present study, we confirmed that body weight gain over 30 years in the early and middle adulthood were significantly associated with later life diabetes risk, independent of the baseline and the early life (in 20s) BMI.

Previous study showed an inverse linear relation was found between BMI and age at diabetes onset (22). Adults with early diagnosed diabetes were more obese and more likely to be female than those with a later onset of T2D. Weight gain in early rather than middle-to-late adulthood played an important role in developing diabetes (23). The relative risk of T2D for a 5-kg m^−2^ increment in BMI was 3.07 for early weight gain (in 18–24s), and 2.12 for late weight-gain (in their 50s) (23). Our results were consistent with the previous studies that investigated the associations of weight change at different period with the risk of T2D (24). In the EPIC-Potsdam study, it was suggested that severe weight gain between ages 25 and 40 y was more strongly associated with risk of diabetes in men (1.5 times) and in women (4.3 times) than weight gain in later life. In addition, greater weight gain, particularly in early adulthood, may be more likely to lead to a lower age at onset of diabetes in men (5 y) and in women (3 y) (24). Though the definition of early adulthood phase was different in our study and other similar studies, it provides us very impressive evidence that the early life, from the 20 to 30 s years, weight gain exerted more prominent effect on risk of earlier developing diabetes.

One possible explanation is that early adulthood marks the end of a critical period for the development of metabolism (25), such as androgen-induced intra-abdominal fat deposition during adolescence may contribute to subsequent hepatic insulin resistance that, in turn, lead to glucose intolerance (26). Individuals who showed weight gain in early life may differ from those who gained weight in later life in behavior and genetic predisposition that may affect metabolism and disease risk (27). Moreover, weight gain in early life tends to be metabolic disturbances reflected by HbA1c, high density lipoprotein cholesterol, high sensitive C-reactive protein, and adiponectin (28).

Several limitations should be acknowledged. Firstly, the early and middle life body weight data was self-reported, which might yield recall bias. The recall bias should be cautiously concerned in this study. However, several previous studies have characterized the validity of retrospective-recalled self-reported weight and measured weight in longitudinal cohorts (29–32), and provided evidence that self-reported weight is reasonably valid surrogate in epidemiology studies (32). In a subgroup of younger women aged 25 - 42 years in the Nurses’ Health Study II, the self-reported and measured weights were highly correlated: the correlation between measured and recalled weight at 18 years was 0.87 (30), and as well in middle aged and elderly study participants: 0.97 for men aged 40-75 years and for women aged 41-65 years (29). In the longitudinal Charleston Heart Study, in elderly study participants aged 60 to 100 years, correlations between reported and measured weights were 0.979 for current, 0.935 for 4-year, and 0.822 for 28-year recall (31). In the present analysis, the average age at baseline were 58.8 years (interquartile range 53.9-62.6). We compared the self-reported body weight at 60 years and the weight measured at baseline examination, the correlation was 0.84 for BMI (kg/m^2^) and 0.85 for body weight (kg). We could not provide the exact valid data on the early life stage. In one of our ongoing community- based cohort studies at Shanghai local area, we assessed the correlation of two repeated recalled body weight during early life to middle-aged and elderly life with a 5-year interval. We found that the two recalled body weight at 50 years were highly correlated (r = 0.72, n = 2,746), and moderately correlated for body weight at 20 years (r = 0.57, n = 2,783).

Secondly, we carefully excluded the missing values on the early to middle life stage in at least one time points. This procedure could yield solid results, along with some missing value bias. However, the relative homogeneity of the cohort in educational attainment and so on actually may serve to enhance the internal validity of this study. Moreover, we conducted the sensitivity analysis by using multiple imputation methods. Because of a large proportion of missing data in recalled weights, we increased the number of imputations (n of impute = 50) to achieve reasonable statistical efficiency. Additionally, when we selected the predictors of diabetes, we imputed not only the main exposure but also all the covariates that were input in the analysis models. The results yielded from the multiple imputation method consistently supported the main analysis.

In addition, the values of age, body weight and height in the present study were shown as means and standard deviation with one decimal place. As the values of weight and height were usually rounded during the empirical work, it should be cautious in the data interpretation.

## Conclusions

Weight gain from early to middle life was linearly and significantly associated with increased risk of incident diabetes. The earlier life weight gain the more strong effect was found on earlier developing diabetes; however, for the risk of developing diabetes after 60 years, each-decade weight gain over the adulthood showed similar effect. It provided evidence that people should pay equal attention to the body weight during the life span, not only after the middle life but also the early stage. More well designed and longitudinal cohort studies with validated early life body weight records or measurements and well documented diseases outcomes are warranted to replicate the present study.

## Data Availability Statement

The original contributions presented in the study are included in the article/**Supplementary Material**. Further inquiries can be directed to the corresponding authors.

## Ethics Statement

The studies involving human participants were reviewed and approved by Medical Ethics Committee of Ruijin Hospital, Shanghai Jiao-Tong University School of Medicine. The patients/participants provided their written informed consent to participate in this study.

## Author Contributions

WW, JL, GN, and MX conceived and designed the study. MX, YQ, ML, YX, JL, TW, and ZZ analyzed data. QW, FS, ZG, GC, JZ, LC, LS, RH, ZY, XT, QS, GQ, GW, ZL, YQ, YH, QL, YZ, YC, CL, YM, YW, SWu, TY, LC, XY, LY, and HD collected data. All authors were involving in writing and revising the manuscript and had final approval of the submitted and published versions. WW, JL and GN are the guarantors of this work and, as such, had full access to all the data in the study and take responsibility for the integrity of the data and the accuracy of the data analysis. All authors contributed to the article and approved the submitted version.

## Funding

This work was funded by the National Natural Science Foundation of China (81941017, 81930021, 81970706, 81970728, 91857205, and 82088102), and the Clinical Research Plan of SHDC (SHDC2020CR1001A and SHDC2020CR3064B), and the Shanghai Municipal Education Commission–Gaofeng Clinical Medicine Grant Support (20152508 Round 2). MX, JL, ML, TW, YX, ZZ, YB, WW, and GN are members of innovative research team of high-level local universities in Shanghai.

## Conflict of Interest

The authors declare that the research was conducted in the absence of any commercial or financial relationships that could be construed as a potential conflict of interest.

## Publisher’s Note

All claims expressed in this article are solely those of the authors and do not necessarily represent those of their affiliated organizations, or those of the publisher, the editors and the reviewers. Any product that may be evaluated in this article, or claim that may be made by its manufacturer, is not guaranteed or endorsed by the publisher.
