# Supplementary material for: The Relative Body Weight Gain From Early to Middle Life Adulthood Associated With Later Life Risk of Diabetes: A Nationwide Cohort Study
Source: Front Endocrinol (Lausanne). 2022 Jul 19;13:927067. doi: 10.3389/fendo.2022.927067 (PMC9343618; doi:10.3389/fendo.2022.927067)
Supplement: Supplementary file 1 [file Table_1.docx]

**Supplementary Materials**

Min Xu, Yan Qi, Gang Chen, et al. The Relative Body Weight Gain from Early to Middle Life Adulthood Associated with Later Life Risk of Diabetes: a Nationwide Cohort Study

**Supplementary Table S1.** The age and sex distribution of the participants according to the seven general geographic regions of China.

**Supplementary Table S2.** Univariate and multivariate of predicators of incident diabetes.

**Supplementary Table S3.** Characteristics of the participants by their body mass index at 20 years and body mass index at baseline categories.

**Supplementary Table S4.** Scientific literature of similar research works.

**Supplementary Table S1.** The age and sex distribution of the participants according to the seven general geographic regions of China.

|  | Total | | Age (years) | | Men | | Women | |
| --- | --- | --- | --- | --- | --- | --- | --- | --- |
| Region | n | % | Means | SD | n | % | n | % |
| Northeast | 6496 | 18.2 | 59.3 | 6.9 | 1696 | 26.1 | 4800 | 73.9 |
| North | 2853 | 8.0 | 57.5 | 6.2 | 950 | 33.3 | 1903 | 66.7 |
| Northwest | 3413 | 9.6 | 59.0 | 6.3 | 915 | 26.8 | 2498 | 73.2 |
| East | 11906 | 33.4 | 58.2 | 6.3 | 3610 | 30.3 | 8296 | 69.7 |
| Central | 3912 | 11.0 | 60.6 | 7.3 | 1231 | 31.5 | 2681 | 68.5 |
| South | 4167 | 11.7 | 57.9 | 6.5 | 1137 | 27.3 | 3030 | 72.7 |
| Southwest | 2864 | 8.0 | 60.2 | 6.9 | 783 | 27.3 | 2081 | 72.7 |

Data are means, standard deviation (SD) for age at baseline, and number (n), percentage (%) for total participants and sex distribution. In the present analysis, Helongjiang, Jilin and Liaoning province were included in northeast China; Beijing was included in north China; Gansu was included in northwest China; Shandong, Shanghai, Zhejiang, Fujian and Jiangxi were included in east China; Henan and Hubei were included in central China; Guangxi and Guangdong were included in south China; and Sichuang and Guizhou were included in southwest China.

**Supplementary Table S2.** Univariate and multivariate of predicators of incident diabetes.

| Parameter |  | Univariate model | | | | Multivariate model | | | |
| --- | --- | --- | --- | --- | --- | --- | --- | --- | --- |
|  |  | Hazard Ratio | 95% Confidence Interval | | *P1* | Hazard Ratio | 95% Confidence Interval | | *P2* |
| Age, years |  | 1.010 | 1.005 | 1.016 | 0.0001 | 1.014 | 1.008 | 1.020 | <.0001 |
| Sex, male = 1, female =2 |  | 0.861 | 0.795 | 0.933 | 0.0002 | 0.905 | 0.794 | 1.032 | 0.1360 |
| BMI, kg/m^2^ |  | 1.059 | 1.051 | 1.067 | <.0001 | 1.034 | 1.020 | 1.049 | <.0001 |
| Height, cm |  | 1.001 | 0.996 | 1.006 | 0.7392 | 0.991 | 0.984 | 0.999 | 0.0330 |
| BMI at 20 years age, kg/m^2^ |  | 1.017 | 1.004 | 1.029 | 0.0083 | 1.006 | 0.989 | 1.024 | 0.4759 |
| Quintiles of physical activity |  | 0.981 | 0.954 | 1.008 | 0.1681 | 0.983 | 0.955 | 1.011 | 0.2194 |
| Quintiles of sedentary time |  | 1.012 | 0.986 | 1.039 | 0.3606 | 1.010 | 0.983 | 1.038 | 0.4632 |
| Current smoker, yes |  | 1.071 | 1.027 | 1.117 | 0.0015 | 1.029 | 0.973 | 1.090 | 0.3151 |
| Former smoker, yes |  | 1.013 | 0.944 | 1.087 | 0.7216 | 0.938 | 0.864 | 1.019 | 0.1309 |
| Alcohol intake, g/day |  | 1.099 | 1.050 | 1.150 | <.0001 | 1.074 | 1.016 | 1.135 | 0.0123 |
| High school and above, yes |  | 1.125 | 1.042 | 1.215 | 0.0027 | 1.118 | 1.027 | 1.217 | 0.0100 |
| Diabetes family history, yes |  | 1.251 | 1.127 | 1.388 | <.0001 | 1.262 | 1.129 | 1.412 | <.0001 |
| Weight gain from 20 to 30 years, kg/m^2^ |  | 1.069 | 1.048 | 1.089 | <.0001 | 1.055 | 1.029 | 1.081 | <.0001 |
| Weight gain from 30 to 40 years, kg/m^2^ |  | 1.066 | 1.045 | 1.087 | <.0001 | 1.038 | 1.012 | 1.064 | 0.0036 |
| Weight gain from 40 to 50 years, kg/m^2^ |  | 1.070 | 1.049 | 1.092 | <.0001 | 1.043 | 1.017 | 1.069 | 0.0009 |

Data are hazard ratio and 95% confidence interval. P values were from the univariate or multivariate Cox regression models. In the multivariate model, all the variables listed in the table were in the model simultaneously.

**Supplementary Table S3.** Characteristics of the participants by their body mass index at 20 years and body mass index at baseline categories.

|  | **Men** | | | | | **Women** | | | |
| --- | --- | --- | --- | --- | --- | --- | --- | --- | --- |
|  | Variable | n | Mean ± SD |  | Median  (Interquartile range) | n. | Mean ± SD |  | Median (Interquartile range) |
| **BMI at 20 years** | |  | | | |  |  |  |  |
|  | |  | | | |  |  |  |  |
| < 21 kg/m^2^,  n=17330 | Age, years | 4573 | 59.5 ± 6.8 |  | 58.6 (54.3-63.6) | 12757 | 57.6 ± 6.2 |  | 56.4 (53.0-61.1) |
|  | BMI, kg/m^2^ |  | 23.8 ± 3.1 |  | 23.7 (21.7-25.8) |  | 23.8 ± 3.2 |  | 23.5 (21.7-25.7) |
|  | Height, cm |  | 167.8 ± 6.2 |  | 168.0 (163.6-172.0) |  | 157.5 ± 5.4 |  | 155.7 (154.0-161.0) |
|  | BMI at 20 years, kg/m^2^ |  | 19.3 ± 1.3 |  | 19.6 (18.6-20.3) |  | 19.0 ± 1.3 |  | 19.2 (18.1-20.1) |
|  | Weight gain from 20 to 30 years, kg/m^2^ |  | 1.3 ± 1.5 |  | 1.0 (0.3-1.9) |  | 1.7 ± 1.8 |  | 1.3 (0.4-2.2) |
|  | Weight gain from 30 to 40 years, kg/m^2^ |  | 1.3 ± 1.7 |  | 0.9 (0.0-1.8) |  | 1.3 ± 1.7 |  | 0.9 (0.0-2.0) |
|  | Weight gain from 40 to 50 years, kg/m^2^ |  | 1.2 ± 1.8 |  | 0.9 (0.0-1.9) |  | 1.3 ± 1.8 |  | 1.0 (0.0-2.1) |
|  |  |  |  |  |  |  |  |  |  |
| ≥ 21 kg/m^2^,  n=17175 | Age, years | 5464 | 60.5 ± 7.1 |  | 59.4 (55.1-65.1) | 11711 | 59.2 ± 6.7 |  | 58.1 (54.2-63.2) |
|  | BMI, kg/m^2^ |  | 25.4 ± 3.5 |  | 25.2 (23.2-27.3) |  | 25.5 ± 3.7 |  | 25.2 (23.1-28.5) |
|  | Height, cm |  | 165.3 ± 6.4 |  | 165.5 (161.3-169.0) |  | 155.1 ± 6.1 |  | 155.0 (151.0-159.1) |
|  | BMI at 20 years, kg/m^2^ |  | 23.1 ± 2.1 |  | 22.6 (21.8-23.9) |  | 23.5 ± 2.5 |  | 22.9 (21.9-24.4) |
|  | Weight gain from 20 to 30 years, kg/m^2^ |  | 0.6 ± 1.3 |  | 0.4 (0.0-1.4) |  | 0.5 ± 2.0 |  | 0.4 (0.0-1.7) |
|  | Weight gain from 30 to 40 years, kg/m^2^ |  | 0.7 ± 1.6 |  | 0.4 (0.0-1.5) |  | 0.7 ± 1.7 |  | 0.4 (0.0-1.5) |
|  | Weight gain from 40 to 50 years, kg/m^2^ |  | 0.6 ± 1.6 |  | 0.4 (0.0-1.5) |  | 0.7 ± 1.7 |  | 0.4 (0.0-1.6) |
|  |  |  |  |  |  |  |  |  |  |
|  | | n | Mean ± SD |  | Median  (Interquartile range) | n | Mean ± SD |  | Median (Interquartile range) |
| **BMI at baseline** | |  |  |  |  |  |  |  |  |
| < 25 kg/m^2^  n=19867 | Age, years | 5620 | 60.2 ± 7.1 |  | 59.2 (54.8-64.5) | 14247 | 58.1 ± 6.4 |  | 56.8 (53.4-61.6) |
|  | BMI, kg/m^2^ |  | 22.4 ± 1.8 |  | 22.7 (21.2-23.9) |  | 22.3 ± 1.8 |  | 22.6 (21.2-23.8) |
|  | Height, cm |  | 166.2 ± 6.3 |  | 166.0 (162.0-170.0) |  | 156.6 ± 5.6 |  | 156.5 (153.0-160.2) |
|  | BMI at 20 years, kg/m^2^ |  | 20.9 ± 2.3 |  | 20.8 (19.4-22.2) |  | 20.6 ± 2.8 |  | 20.3 (18.7-22.2) |
|  | Weight gain from 20 to 30 years, kg/m^2^ |  | 0.6 ± 1.3 |  | 0.4 (0.0-1.2) |  | 0.7 ± 1.8 |  | 0.5 (0.0-1.7) |
|  | Weight gain from 30 to 40 years, kg/m^2^ |  | 0.5 ± 1.4 |  | 0.4 (0.0-1.1) |  | 0.6 ± 1.5 |  | 0.4 (0.0-1.2) |
|  | Weight gain from 40 to 50 years, kg/m^2^ |  | 0.4 ± 1.4 |  | 0.3 (0.0-1.0) |  | 0.5 ± 1.5 |  | 0.4 (0.0-1.3) |
|  |  |  |  |  |  |  |  |  |  |
| ≥ 25 kg/m^2^,  n=14626 | Age, years | 4414 | 59.8 ± 6.9 |  | 58.8 (54.6-64.2) | 10212 | 58.8 ± 6.6 |  | 57.8 (53.8-62.7) |
|  | BMI, kg/m2 |  | 27.6 ± 2.5 |  | 27.0 (25.9-28.6) |  | 27.8 ± 2.8 |  | 27.1 (26.0-28.8) |
|  | Height, cm |  | 166.8 ± 6.6 |  | 167.0 (162.8-171.0) |  | 156.0 ± 6.1 |  | 156.0 (152.0-160.0) |
|  | BMI at 20 years, kg/m^2^ |  | 22.1 ± 2.9 |  | 21.8 (20.3-23.6) |  | 21.9 ± 3.0 |  | 21.7 (19.9-23.7) |
|  | Weight gain from 20 to 30 years, kg/m^2^ |  | 1.4 ± 1.6 |  | 1.1 (0.3-1.9) |  | 1.7 ± 2.0 |  | 1.6 (0.4-2.3) |
|  | Weight gain from 30 to 40 years, kg/m^2^ |  | 1.6 ± 1.8 |  | 1.4 (0.4-2.3) |  | 1.5 ± 1.8 |  | 1.3 (0.4-2.3) |
|  | Weight gain from 40 to 50 years, kg/m^2^ |  | 1.5 ± 1.9 |  | 1.4 (0.3-2.3) |  | 1.6 ± 1.9 |  | 1.3 (0.4-2.3) |

**Supplementary Table S4.** Scientific literature of similar research works

| **Author** | **Title** | **References** | **Study population** | **Exposure** | **Outcomes** | **Main Findings** |
| --- | --- | --- | --- | --- | --- | --- |
| Tirosh A, Shai I, Afek A, Dubnov-Raz G | Adolescent BMI Trajectory and Risk of Diabetes versus Coronary Disease | N Engl J Med. 2011;364(14):1315-25. | 37,674 apparently healthy young men of Israeli Army Medical Corps | Deciles of BMI at 17 years of age； | 650,000 person-years of follow-up (mean follow-up, 17.4 years), documented 1173 incident cases of type 2 diabetes and 327 of angiography-proven coronary heart disease | In multivariate models adjusted for age, family history, blood pressure, lifestyle factors, and biomarkers in blood, elevated adolescent BMI was a significant predictor of both diabetes (HR for the highest vs. the lowest decile, 2.76; 95% [CI], 2.11-3.58) and angiography-proven coronary heart disease (HR, 5.43; 95% CI, 2.77-10.62). Further adjustment for BMI at adulthood completely ablated the association of adolescent BMI with diabetes (HR 1.01; 95% CI, 0.75 to 1.37) but not the association with coronary heart disease (HR, 6.85; 95% CI, 3.3- 14.21). The risk of diabetes is mainly associated with increased BMI close to the time of diagnosis, the risk of coronary heart disease is associated with an elevated BMI both in adolescence and in adulthood, supporting the hypothesis that the processes causing incident coronary heart disease, particularly atherosclerosis, are more gradual than those resulting in incident diabetes. |
| de Lauzon-Guillain B, Balkau B, Charles MA, et al. | Birth weight, body silhouette over the life course, and incident diabetes in 91,453 middle-aged women from the French Etude Epidemiologique de Femmes de la Mutuelle Generale de l'Education Nationale (E3N) Cohort | Diabetes Care. 2010;33(2):298-303. | E3N is a cohort study of French women born in 1925-1950 and followed by questionnaire every 2 years. At baseline, in 1990, women were asked to report their current weight, height, and body silhouette at various ages. Birth weight was recorded in 2002. | Birth weight and body silhouette at 8 years, at menarche, in young adulthood (20-25 years), and in mid-adulthood (35-40 years). | Of the 91,453 women who were nondiabetic at baseline, 2,534 developed diabetes over the 15 years of follow-up. Cases of diabetes were self-reported or obtained by drug reimbursement record linkage and further validated | Birth weight and body silhouette at 8 years, at menarche, and in young adulthood (20-25 years) were inversely associated with the risk of diabetes, independently of adult BMI during follow-up (all P(trend) < 0.001). In mid-adulthood (35-40 years), the association was reversed, with an increase in risk related to a larger body silhouette. An increase in body silhouette from childhood to mid-adulthood amplified the risk of diabetes. |
| de Mutsert R, Sun Q, Willett WC, et al. | Overweight in early adulthood, adult weight change, and risk of T2D, cardiovascular diseases, and certain cancers in men: A cohort study | Am J Epidemiol. 2014;179(11):1353-65. | 39,909 male participants of the Health Professionals Follow-Up Study who were 40-75 years of age in 1986 and were followed until 2008 | BMI at 21 years; adult weight gain. Adult weight change was calculated as the difference between the reported weight at the baseline of the study in 1986 and the recalled weight at the age of 21 years. | 8,755 incident cases of obesity-related chronic diseases (type 2 diabetes mellitus, cardiovascular diseases, and colorectal, renal, pancreatic, and esophageal cancers) | Compared with a BMI at 21 years of 18.5-22.9, the composite hazard ratio for a BMI of 23-24.9 was 1.22 (95% confidence interval (CI): 1.16, 1.29), that for a BMI of 25.0-27.4 was 1.57 (95% CI: 1.48, 1.67), that for a BMI of 27.5-29.9 was 2.40 (95% CI: 2.17, 2.65), and that for a BMI ≥30.0 was 3.15 (95% CI: 2.76, 3.60). The composite hazard ratios for adult weight gain compared with a stable weight were 1.12 (95% CI: 1.03, 1.22) for a gain of 2.5-4.9 kg, 1.41 (95% CI: 1.31, 1.52) for a gain of 5-9.9 kg, 1.72 (95% CI: 1.59, 1.86) for a gain of 10-14.9 kg, and 2.45 (95% CI: 2.27, 2.63) for a gain ≥15 kg. Adiposity in early adulthood and adult weight gain were both associated with marked increases in the risk of major chronic diseases in middle-aged and older men, and these associations were already apparent at modest levels of overweight and weight gain. |
| Black E, Holst C, Astrup A, et al. | Long-term influences of body-weight changes, independent of the attained weight, on risk of impaired glucose tolerance and T2D | Diabet Med. 2005;22(9):1199-205. | A longitudinal study of two cohorts: one of juvenile-onset obese (n = 248) and one of randomly selected control (n = 320) men, weighed at average ages of 20, 33, 44 and 51 years, respectively | Weight gain since age 20. The first analysis addressed the effects of BMI change between age 20 and age 51 and the second series the effects of BMI changes during the intermediate intervals. | The prevalence of IGT and type 2 diabetes. | The risk of IGT was higher the greater the weight gain since age 20 (odds ratio of 1.10 per unit kg/m2 of BMI gain, 95% CI 1.03-1.17, P = 0.004), and weight gain during both the early and later ages contributed to the increased risk. Obese men, maintaining weight since age 20, had lower risk of IGT than non-obese men who became similarly obese by age 51. The risk of type 2 diabetes increased by weight gain in early adult life, but not by more recent weight gain in the later periods. |
| Zheng Y, Manson JE, Yuan C, et al. | Associations of Weight Gain From Early to Middle Adulthood With Major Health Outcomes Later in Life | JAMA. 2017;318(3):255-269. | Cohort analysis of US women from the Nurses' Health Study (1976-June 30, 2012) and US men from the Health Professionals Follow-Up Study (1986-January 31, 2012) who recalled weight during early adulthood (at age of 18 years in women; 21 years in men) and reported current weight during middle adulthood (at age of 55 years). | Weight change from early to middle adulthood (age of 18 or 21 years to age of 55 years). | Cardiovascular disease, cancer, and death were confirmed by medical records or the National Death Index. A composite healthy aging outcome was defined as being free of 11 chronic diseases and major cognitive or physical impairment. | For type 2 diabetes, the adjusted incidence per 100 000 person-years was 207 among women who gained a moderate amount of weight (≥2.5 kg to <10 kg) vs 110 among women who maintained a stable weight (weight loss ≤2.5 kg or gain <2.5 kg) (absolute rate difference [ARD] per 100 000 person-years, 98; 95% CI, 72 to 127) . The multivariable-adjusted odds ratio for the composite healthy aging outcome associated with moderate weight gain was 0.78 (95% CI, 0.72 to 0.84) in women and 0.88 (95% CI, 0.79 to 0.97) in men. Higher amounts of weight gain were associated with greater risks of major chronic diseases and lower likelihood of healthy aging. |
| Sun W, Shi L, Ye Z, et al. | Association Between the Change in Body Mass Index from Early Adulthood to Midlife and Subsequent T2D Mellitus | Obesity (Silver Spring). 2016;24(3):703-9. | This study included 120,666 middle-aged and elderly, whose retrospectively self-reported body weight at 20 and 40 years and measured height were available. | BMI at 20 and 40 years and BMI change in between were defined as early-adulthood BMI, midlife BMI, and early-adulthood BMI change. | Presence of type 2 diabetes mellitus (T2DM) after midlife. | The odds ratio (OR) for T2DM associated with each 1-unit increment of early-adulthood or midlife BMI was 1.08 (95% confidence interval (CI), 1.07-1.08) and 1.09 (95% CI, 1.09-1.10) respectively. In the cross-tabulation of both early-adulthood BMI and BMI change, the prevalence of T2DM increased across both variables. Compared with participants with normal early-adulthood weight and BMI increase/decrease ≤1, the OR (95% CI) for T2DM of participants with early-adulthood overweight/obesity and BMI increase ≥4 kg/m2 was 3.49 (3.05-4.00). For participants with early-adulthood underweight and BMI increase/decrease ≤ 1, the OR (95% CI) was 0.85 (0.75-0.97). Subgroup analysis according to sex and age showed similar trends. |
| Kodama S, Horikawa C, Fujihara K, et al. | Quantitative relationship between body weight gain in adulthood and incident T2D: a meta-analysis | Obes Rev. 2014;15(3):202-14 | This meta-analysis quantified the risk of type 2 diabetes mellitus (T2DM) preceded by body weight (BW) gain in the general population. | The body weight gain was divided into early weight-gain, which was defined as BW gain from early adulthood (18-24 years of age) to cohort entry (≥25 years of age), and late weight-gain, which was defined as BW gain from cohort entry. | Incident type 2 diabetes | The pooled relative risk (RR; 95% confidence interval [CI]) of T2DM for an increment of BW gain standardized into a 5-kg m(-2) increment in BMI was 3.07 (2.49-2.79) for early weight-gain and 2.12 (1.74-2.58) for late weight-gain. When limiting analysis to studies that concurrently examined T2DM risk for current BMI, a larger magnitude of T2DM risk was revealed for early weight-gain compared with current BMI (RR [95% CI], 3.38 [2.20-5.18] vs. 2.39 [1.58-3.62]), while there was little difference between late weight-gain (RR [95% CI], 2.21 [1.91-2.56]) and current BMI (RR [95% CI], 2.47 [1.97-3.30]). |
| Montonen J, Boeing H, Schleicher E, et al. | Association of changes in body mass index during earlier adulthood and later adulthood with circulating obesity biomarker concentrations in middle-aged men and women | Diabetologia. 2011;54(7):1676-83. | The study included 1,612 participants from the European Prospective Investigation into Cancer and Nutrition (EPIC)-Potsdam Study. | BMI changes based on recalled BMI for the age ranges 25-40 years (earlier adulthood) and 40-55 years (later adulthood) | Circulating obesity biomarkers in middle age: HbA(1c), ALT, GGT, HDL-C, hs-CRP, total adiponectin concentration | BMI changes during both time periods as well as BMI at age 25 years were significantly associated with circulating levels of adiponectin, γ-glutamyltransferase (GGT), alanine aminotransferase (ALT), high-sensitivity C-reactive protein (hs-CRP) and HDL-cholesterol (HDL-C) in both sexes, and of HbA(1c) in women. However, BMI gain for the age range 25-40 years was significantly more strongly associated with unfavourable levels of adiponectin, hs-CRP, HDL-C and HbA(1c) in men and women, and of GGT and ALT in men (p difference <0.05) than BMI gain for the age range 40-55 years. The results support the hypothesis that an increase in BMI in earlier adulthood is more strongly associated with unfavourable circulating levels of obesity biomarkers later in life than is an increase in BMI in later adulthood. |
| Zhu Y, Zheng R, Hu C, et al. | Association of early adulthood weight and subsequent weight change with cardiovascular diseases: Findings from REACTION study | Int J Cardiol. 2021;332:209-215. | This study included 121160 participants in a large population-based cohort in China. | Body weight at 20 and 40 years of age wase self-reported. Weight change from early adulthood to midlife was defined as weight at 40 years of age minus that at 20 | Reported total CVD events (CHD, stroke, or MI) | The odds ratios (ORs) were 1.20 (95% CI, 1.10-1.31) for coronary heart disease (CHD), 1.74 (95% CI, 1.36-2.22) for myocardial infarction (MI), 1.14 (95% CI, 0.99-1.32) for stroke and 1.21 (95% CI, 1.12-1.31) for total CVD among individuals with early overweight and became more prominent for early obesity. Meanwhile, A moderate weight gain of 2.5 kg between early adulthood and midlife significantly increased the risk of CHD (OR: 1.18, 95% CI: 1.05-1.32), stroke (OR: 1.19, 95% CI: 1.03-1.38) and total CVD (OR: 1.15, 95% CI: 1.04-1.27), and the risk escalated with higher amounts of weight gain. Conversely, a weight loss of 2.5 kg conferred lower risk of CVD compared with a stable weight. |
